# Measuring adherence to antihypertensive medication using an objective test in older adults attending primary care: cross-sectional study

**DOI:** 10.1038/s41371-021-00646-w

**Published:** 2021-12-07

**Authors:** James P. Sheppard, Ali Albasri, Pankaj Gupta, Prashanth Patel, Kamlesh Khunti, Una Martin, Richard J. McManus, F. D. Richard Hobbs

**Affiliations:** 1 Nuffield Department of Primary Care Health Sciences, University of Oxford, Oxford, UK; 2 Department of Chemical Pathology and Metabolic Diseases, University Hospitals of Leicester NHS Trust, Leicester, UK; 3 Department of Cardiovascular Sciences, University of Leicester, Leicester, UK; 4 Birmingham Medical School, College of Medical and Dental Sciences, University of Birmingham, Birmingham, UK

**Keywords:** Preventive medicine, Risk factors

## Abstract

Analysis of urine samples using liquid chromatography-tandem mass spectrometry (LC-MS/MS) has previously revealed high rates of non-adherence to antihypertensive medication. It is unclear whether these rates represent those in the general population. This study aimed to investigate whether it is feasible to collect urine samples in a primary care setting and analyse them using LC-MS/MS to detect non-adherence to antihypertensive medication. This study used a prospective, observational cohort design. Consecutive patients were recruited opportunistically from five general practices in UK primary care. They were aged ≥65 years with hypertension and had at least one antihypertensive prescription. Participants were asked to provide a urine sample for analysis of medication adherence. Samples were sent to a laboratory via post and analysed using LC-MS/MS. Predictors of adherence to medication were explored with multivariable logistic regression. Of 349 consecutive patients approached for the study, 214 (61.3%) gave informed consent and 191 (54.7%) provided a valid urine sample for analysis. Participants were aged 76.2 ± 6.6 years and taking a median of 2 antihypertensive medications (IQR 1–3). A total of 27/191 participants (14.2%) reported not taking all of their medications on the day of urine sample collection. However, LC-MS/MS analysis of samples revealed only 4/27 (9/191 in total; 4.7%) were non-adherent to some of their medications. Patients prescribed more antihypertensive medications were less likely to be adherent (OR 0.24, 95%CI 0.09–0.65). Biochemical testing for antihypertensive medication adherence is feasible in routine primary care, although non-adherence to medication is generally low, and therefore widespread testing is not indicated.

## Introduction

The population is ageing [1] and the number of people living with age-related chronic conditions is increasing [2]. This is accompanied by more prescriptions of long-term medications. More than one in three adults aged ≥75 years are prescribed five or more medications [3], a situation known as polypharmacy [4]. Polypharmacy is associated with partial or non-adherence to medications, particularly those prescribed for the prevention of disease [5–7]. Non-adherence to medications used for the prevention of cardiometabolic diseases is important since this can significantly increase an individual’s risk of future events [8, 9]. However, identifying non-adherence can be difficult [10]. Information about an individual’s medication adherence can be captured through direct questioning [11], questionnaires [12, 13], directly observed dosing [14], prescription refill data, pill counts, and electronic ‘event’ monitoring [11], but used in isolation, these approaches have limitations which affect their ability to accurately confirm medication adherence in routine clinical practice [15, 16].

New methods for identifying non-adherence now exist using liquid chromatography-tandem mass spectrometry (LC-MS/MS) to detect a number of medications measurable in an individual’s urine [17]. This approach has revealed high rates (25–42%) of previously unrecognised non-adherence to medication in hypertensive patients [6, 17]. However, most previous studies utilising this approach have enroled complex hypertensive patients from clinics where individuals have been referred due to suspected non-adherence or suboptimal blood pressure control [6, 17, 18]. It is unclear whether the medication adherence rates seen in these complex patients are representative of those in the general population presenting in primary care, or even whether patients would be willing to provide urine samples for assessment of adherence in this setting. The present study, therefore, aimed to investigate whether it is feasible to collect urine samples in a primary care setting and analyse them using the LC-MS/MS method to measure adherence to antihypertensive medication.

## Methods

### Study design

This study used a prospective, observational cohort design, enroling participants in primary care aged ≥65 years with hypertension and prescribed at least one blood pressure lowering medication. Patients attending the practice were asked to give informed consent and provide a urine sample for researchers to examine whether antihypertensive medications were present in their system. Anonymised data were collected for patients declining to participate in the study for comparison to those who did participate. Ethical approval for this study was given by South Central—Oxford A Research Ethics Committee (ref: 18/SC/0647). Detailed methods are given in the Supplementary appendix.

### Study participants

Consecutive patients aged ≥65 years with an electronic medical record coded diagnosis of hypertension and prescribed at least one blood pressure lowering medication were approached opportunistically. Participating general practices were located in the Thames Valley region of England.

Participating general practitioners (GPs) were asked to identify patients attending routine medication reviews or chronic disease management clinics meeting the eligibility criteria. Consent and sample collection took place directly after each routine appointment to ensure the subsequent assessment of medication adherence was representative of an individual’s true drug-taking behaviour.

### Data collection

For all patients approached to participate in the study, members of the care team extracted anonymised data from their electronic health record, detailing basic patient characteristics and medical history. Data included information relating to patient characteristics, blood pressure, medical history, and all antihypertensive medications prescribed. Prior to collecting the urine sample, all consenting participants were asked the question “*Have you taken all of your blood pressure pills today?*” and responses were categorised as “all medications”, “some medications”, or “no medications”.

### Urine sample collection and analysis

Participants were informed from the outset that the study was investigating the feasibility of collecting urine samples and testing whether patients had taken all of their medications as prescribed. They were reassured that this information would remain entirely confidential and members of the care team would not be made aware of the results of the urine test. A 10 ml urine sample was collected in a standard plastic container immediately after informed consent had been obtained. Each sample was transferred from the clinic site to the laboratory at University Hospitals of Leicester at room temperature, via a post office next day delivery service. We have previously demonstrated that samples remain stable for 72 h after collection [19].

All samples received by the laboratory were stored at −80 °C and then batch analysed at the end of the study. LC-MS/MS was performed to detect all antihypertensive drug classes, using an Agilent Technologies 1200 series High Pressure Liquid Chromatograph interfaced with an Agilent Technologies 6410 Triple Quad Mass Spectrometer fitted with a Jetstream electrospray (ESI) source [17]. The test is a standard laboratory test that is accredited by United Kingdom Accreditation Service (UKAS). All samples were destroyed at the end of the study, after the analysis had been completed.

### Sample size calculation

The study aimed to collect anonymised data from approximately 285 patients, gathering urine samples from at least 200 consenting participants. This assumed 70% [20] of those approached would give informed consent for their samples to be collected and analysed, allowing a recruitment rate of 70% to be estimated with an accuracy of ±6% (95% confidence interval of 64% to 75%). Recruitment of at least 200 participants was estimated to be sufficient to calculate a medication adherence of 75% to within ±7%.

### Statistical analysis

The primary outcome of this study was to determine the proportion of patients attending a routine medication review or check-up in primary care (denominator population) who gave informed consent to provide a urine sample for analysis of medication adherence (numerator). We did not pre-specify feasibility criteria for this study, and so have applied criteria previously described in the literature which defined feasibility as ≥50% of patients agreeing to provide a sample and 95% of collected samples being suitable for analysis [21]. Participant characteristics predicting the likelihood of consent to provide a urine sample were explored using multivariable logistic regression.

Medication adherence was defined as a binary outcome; adherent patients were those in whom all prescribed antihypertensive medications were present in their urine sample. Non-adherent patients were those in whom only some or none of their prescribed medications were detected in the urine sample. Adherence to medication was estimated using descriptive statistics, across the entire study population and sub-grouped by type of medication prescribed and whether blood pressure was controlled (±140/90 mm Hg).

Predictors of non-adherence to medication were explored with logistic regression, including age, sex, blood pressure, co-morbidities, and the number of antihypertensive medications as independent predictor variables. Sensitivity analyses were performed including blood pressure control (±140/90 mm Hg) in the model. Analyses were undertaken for descriptive purposes only, so no attempt was made to reduce the model using selection methods. All analyses were undertaken using STATA version 16.0 (MP edition, StataCorp, College Station, Texas, USA).

## Results

### Population characteristics

**Fig. 1:**
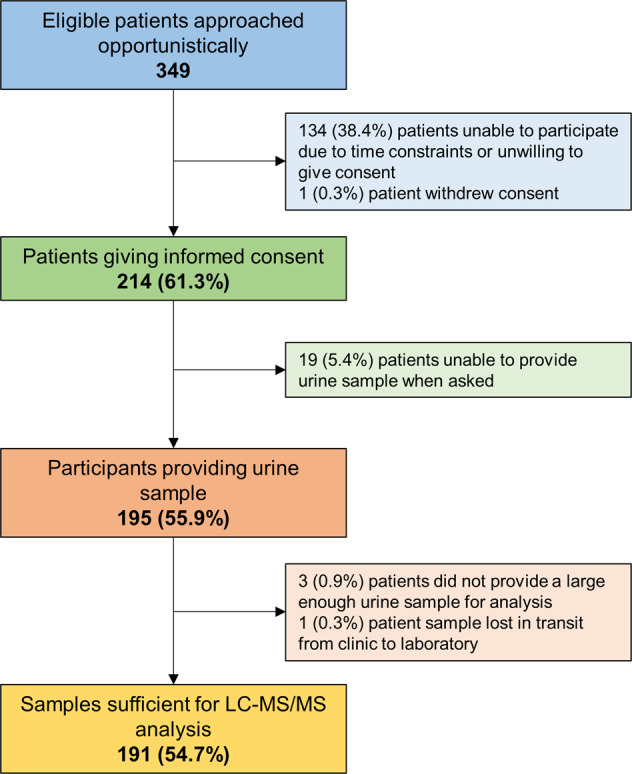
Flow of patients through the study from approach to provision of a urine sample. LC-MS/MS = liquid chromatography-tandem mass spectrometry.

A total of 349 consecutive, eligible patients from five semi-urban general practices in areas of relatively low deprivation were approached for the study. Of these, 214 (61.3%, 95%CI 56.0% to 66.5%) gave informed consent to provide a urine sample for analysis (Fig. 1). The most common reasons for non-participation were a lack of time to attend an additional clinic appointment and provide a sample. One participant withdrew consent.

Patients were aged 76 ± 7 years, with similar proportions of men (171, 49.1%) and women (178, 50.9%), and a mean blood pressure of 135/75 mm Hg (Table 1). Of those giving informed consent, 191 (89.3%, 95% CI 84.3% to 93.1%) were able to provide a sample suitable for analysis and were included in all analyses of medication adherence. Of those not providing a sample suitable for analysis, 19 (5.4%) were unable to produce a urine sample, three (0.9%) provided an insufficient amount (i.e., <10 ml) of urine to enable analysis and one urine sample (0.3%) was lost in transit from the clinic to the laboratory.

**Table 1:** Characteristics of patients consenting/not consenting to provide urine samples for analysis.

Characteristic	All patients		Consenting patients		Non-consenting patients	
	Number	%/SD	Number	%/SD	Number	%/SD
Population size (%)	348	100.0%	214	61.5%	134	38.5%
Age, years; mean (SD)	76.6	6.8	76.2	6.6	77.3	7.1
Sex, female (%)	171	49.1%	95	44.4%	76	56.7%
BMI, kg/m^2^; mean (SD)	28.8	5.4	29.0	5.6	28.6	5.2
Smoking Status*						
Never smoked (%)	108	40.3%	100	46.5%	8	15.1%
Current smoker (%)	43	16.0%	10	4.7%	33	62.3%
Ex-smoker (%)	116	43.3%	104	48.4%	12	22.6%
Systolic BP, mmHg; mean (SD)	134.5	14.7	135.3	15.3	133.4	13.6
Diastolic BP, mmHg; mean (SD)	75.0	9.6	75.0	9.9	75.1	9.1
Controlled BP (<140/90 mm Hg; %)	222	64.0%	133	62.2%	89	66.9%
Co-morbidities						
Myocardial infarction, (%)	22	6.3%	16	7.5%	6	4.5%
Stroke, (%)	32	9.2%	22	10.3%	10	7.5%
Any cardiovascular disease, (%)	94	27.0%	62	29.0%	32	23.9%
Diabetes, (%)	85	24.4%	56	26.2%	29	21.6%
Chronic kidney disease, (%)	76	21.8%	44	20.6%	32	23.9%
Atrial fibrillation, (%)	82	23.6%	47	22.0%	35	26.1%
Arthritis, (%)	121	34.8%	99	46.3%	22	16.4%
Dementia, (%)	5	1.4%	4	1.9%	1	0.7%
Depression, (%)	54	15.5%	35	16.4%	19	14.2%
Multi-morbidity (≥2 conditions; %)†	141	40.5%	97	45.3%	44	32.8%
Antihypertensives prescribed						
ACE inhibitor (%)	159	45.7%	86	40.2%	73	54.5%
Angiotensin II receptor blocker (%)	120	34.5%	75	35.0%	45	33.6%
Calcium channel blocker (%)	160	46.0%	97	45.3%	63	47.0%
Thiazide/thiazide-like diuretic (%)	69	19.8%	32	15.0%	37	27.6%
Beta-blocker (%)	119	34.2%	71	33.2%	48	35.8%
Alpha-blocker (%)	26	7.5%	12	5.6%	14	10.4%
Loop diuretic (%)	67	19.3%	36	16.8%	31	23.1%
Aldosterone antagonist (%)	14	4.0%	10	4.7%	4	3.0%
Other antihypertensive (%)	5	1.4%	4	1.9%	1	0.7%
Number of antihypertensives prescribed						
1 Antihypertensive (%)	108	31.0%	81	37.9%	27	20.1%
2 Antihypertensives (%)	126	36.2%	75	35.0%	51	38.1%
3 Antihypertensives (%)	81	23.3%	41	19.2%	40	29.9%
≥4 Antihypertensives (%)	29	8.3%	16	7.5%	13	9.7%

One participant subsequently withdrew consent, leaving 348 patients with data available for analysis.

*BMI* body mass index, *BP* blood pressure, *ACE* angiotensin converting enzyme.

*Smoking status only available for 53 patients in the non-consenting group. Figures given as a proportion of those with available data.

†Based on the number of conditions present of those collected: Myocardial infarction, stroke, diabetes, chronic kidney disease, atrial fibrillation, arthritis, dementia, and depression.

### Predictors of consent to provide urine samples

**Fig. 2:**
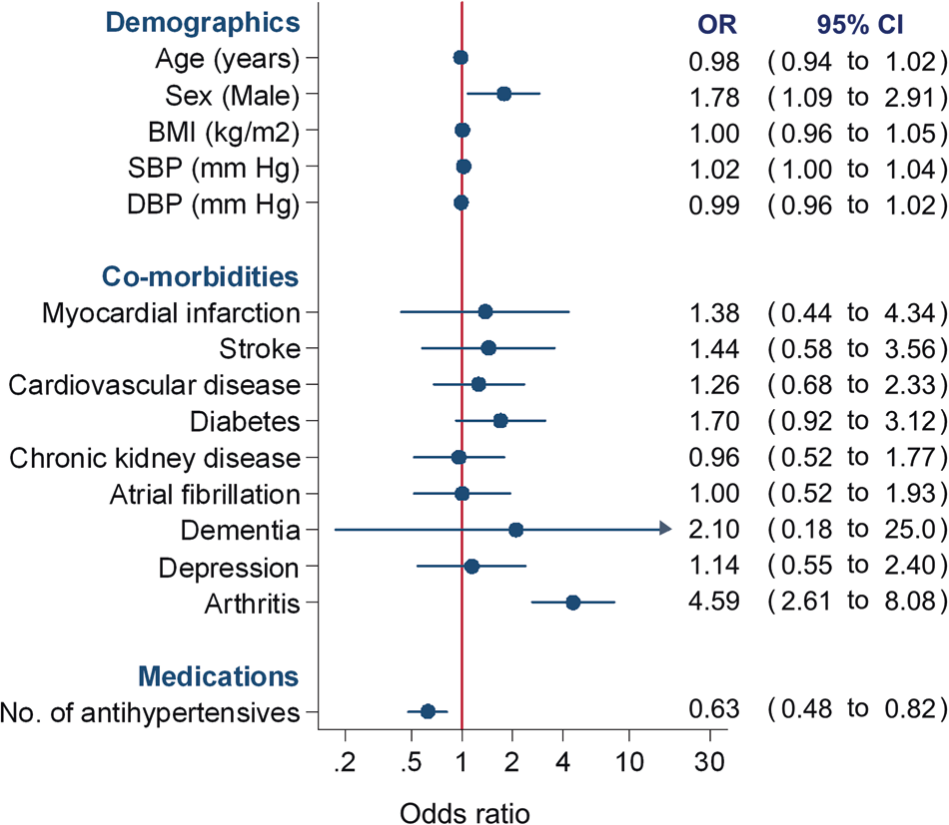
Predictors of patient consent to provide a urine sample for the study. *BMI* body mass index, *SBP* systolic blood pressure, *DBP* diastolic blood pressure; *OR* odds ratio, *CI* confidence interval. Individual drug classes could not be included in the due to collinearity.

In multivariable analyses, being male (Adjusted odds ratio [OR] 1.78, 95%CI 1.09 to 2.91), having a history of arthritis (OR 4.59, 95%CI 2.61 to 8.08) and those with higher systolic blood pressure (OR 1.02, 95%CI 1.00 to 1.04) were more likely to consent to the study, although mean systolic blood pressure was broadly similar between groups (135 vs 133 mm Hg; Table 1). Patients prescribed more antihypertensive medications (OR 0.63, 95%CI 0.48 to 0.82) were less likely to give informed contested for urine sample collection (Fig. 2).

### Adherence to antihypertensive medication

A total of 18 participants (9.4%, 95%CI 5.7% to 14.5%) reported not taking their medications on the day of urine sample collection, and a further 9 (4.7%, 95%CI 2.2% to 8.8%) reported that they had only taken some of their prescribed medications (Table 2). Of the 18 participants stating that they had not taken any medications yet that day, 16 (88.9%, 95%CI 65% to 98.6%) were shown to be adherent by LC-MS/MS and two (11.1%, 95%CI 1.4% to 34.7%) were taking at least one, but not all of their prescribed medications. Of the nine participants stating that they had only taken some of their prescribed medications that day, seven (77.8%, 95% CI 40.9% to 97.2%) were measured as fully adherent and two (22.2%, 95%CI 2.8% to 60.0%) were taking some of their prescribed medications.

**Table 2:** Self-reported and biochemically determined medication adherence by the number of antihypertensives prescribed (*n* = 191).

Number of antihypertensives prescribed	Self-reported adherence						Biochemically determined adherence					
	All medications		At least 1 but not all medications		No medications		All medications		At least 1 but not all medications		No medications	
	Number	%	Number	%	Number	%	Number	%	Number	%	Number	%
Overall^a^	163	85.8%	9	4.7%	18	9.5%	182	95.3%	8	4.2%	1	0.5%
1 Antihypertensive	76	92.7%	0	0.0%	6	7.3%	82	100.0%	0	0.0%	0	0.0%
2 Antihypertensives	49	79.0%	5	8.1%	8	12.9%	59	95.2%	2	3.2%	1	1.6%
3 Antihypertensives^a^	28	84.8%	3	9.1%	2	6.1%	31	91.2%	3	8.8%	0	0.0%
≥4 Antihypertensives	10	76.9%	1	7.7%	2	15.4%	10	76.9%	3	23.1%	0	0.0%

^a^1 Participant did not report whether they had taken their pills that day.

Overall, 182 participants (95.3%, 95%CI 91.2% to 97.8%) were fully adherent to all of their antihypertensive medications, eight (4.2%, 95%CI 1.8% to 8.1%) were partially adherent (i.e., taking at least one, but not all of their medications) and only one participant was entirely non-adherent, despite reporting taking all of their antihypertensives. Loop diuretics were the most commonly prescribed antihypertensive drug class not detected in participant’s urine samples (Table 3).

**Table 3:** Biochemically determined medication adherence by baseline blood pressure and type of antihypertensive prescribed (*n* = 191).

Subgroup	Category	Biochemically determined adherence			
		Detected		Not detected	
		Number	%	Number	%
Blood pressure	Blood pressure controlled (<140/90 mm Hg)	166	97.1%	5	2.9%
	Blood pressure uncontrolled (≥140/90 mm Hg)	66	94.3%	4	5.7%
Antihypertensive prescribed	ACE inhibitors	76	97.4%	2	2.6%
	Angiotensin II receptor blockers	67	98.5%	1	1.5%
	Calcium channel blockers	83	97.6%	2	2.4%
	Thiazide/thiazide-like diuretics	24	92.3%	2	7.7%
	Beta-blockers	58	100.0%	0	0.0%
	Alpha-blockers	10	100.0%	0	0.0%
	Loop diuretics	24	85.7%	4	14.3%
	Aldosterone antagonists	8	100.0%	0	0.0%
	Other antihypertensives	3	100.0%	0	0.0%

### Predictors of adherence to antihypertensive medication

**Fig. 3:**
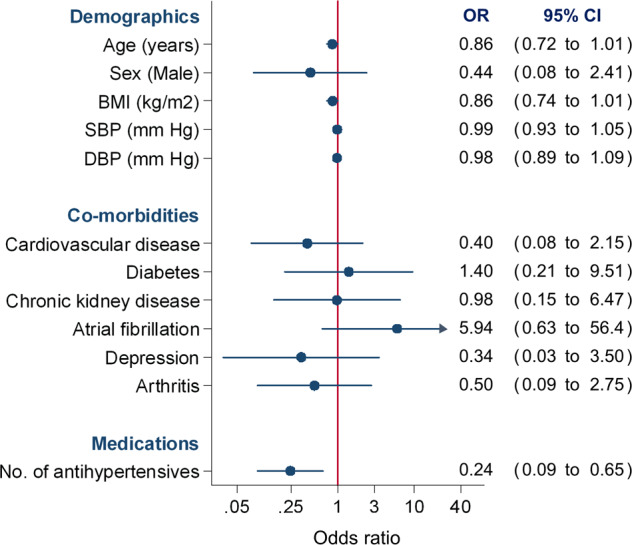
Predictors of complete antihypertensive medication adherence. BMI body mass index, SBP systolic blood pressure, DBP diastolic blood pressure; OR odds ratio, CI confidence interval. An insufficient number of (non-adherence) outcome events were available to include myocardial infarction, stroke, depression as candidate predictors in the model.

In multivariable analyses, patients prescribed more antihypertensive medications (OR 0.24, 95%CI 0.09 to 0.65) were less likely to be adherent to antihypertensive medication (Fig. 3). No other factors predicted non-adherence to antihypertensive medication. Findings were unchanged in sensitivity analyses including blood pressure control as a variable in the model, which was not associated with medication adherence (OR 0.88, 95%CI 0.17 to 4.49).

## Discussion

### Summary of main findings

In this study of 349 hypertensive patients attending primary care for a routine medication review or chronic disease management clinic, almost two thirds (61%) of patients agreed to provide a urine sample to test for the presence of antihypertensive medication, suggesting patients are willing to engage with this method of measuring medication adherence in routine clinical practice. Only 89% of consenting patients were able to provide a urine sample suitable for analysis, which is lower than the 95% of samples considered to indicate the feasibility and may reflect the age of our population [21]. Amongst those 191 participants who consented and were able to provide a sample, self-reported adherence to medication did not appear to be related to biochemically determined adherence. This may have been caused by traces of drugs taken on previous days still being present in the participant’s sample as LC-MS/MS can detect medications for up to 4–6 half-lives in the urine [22]. It may also reflect uncertainty from the patient about when they last took their tablets. Overall, non-adherence to antihypertensive medication was very low (<5%), in contrast to previous studies using this approach [6, 17, 18]. This may be a conservative estimate, given the limitations of the LC-MS/MS method in terms of detecting drugs with longer half-lives, and considering that a third of patients declined to participate in the study, although the characteristics of consenting and non-consenting patients were broadly similar. Given these low rates of non-adherence, widespread testing using this approach should not be recommended in the community.

### Strengths and limitations

The present study included a sample of patients broadly representative of the population approached in primary care, in terms of age, blood pressure, co-morbidities, and antihypertensives prescribed. Patients were recruited from practices of relatively low deprivation and those taking multiple antihypertensive medications were less likely to participate and so caution should be exercised when applying these findings to patients with high deprivation on multiple treatments.

Biochemically determined adherence using the LC-MS/MS method can objectively determine whether a patient has taken a prescribed medication, and so is not prone the same biases that affect other methods for measuring adherence such as self-reported adherence or pill counts [15]. However, this approach only permits the assessment of medication adherence at a single moment in time. As such, it is dependent on the half-life of each individual drug examined, and the ability of the patient’s body to metabolise it. The LC-MS/MS method can detect medications for up to 4–6 half-lives in the urine. Therefore, medications taken on previous days may still have been detected, even if a patient had not taken their tablets on the day of urine sample collection. This could give the impression that an individual was adherent to therapy when in fact they had not taken their medication that day. Indeed, in the present study, there was some discrepancy between the results of the LC-MS/MS analysis and self-reported medication adherence. Thus, we would recommend a multi-method approach to determining medication adherence in routine practice, perhaps combining the LC-MS/MS method with direct questioning [11].

Opportunistic recruitment was necessary in this study to ensure patients did not modify their medication-taking behaviour in anticipation of the urine sample collection. However, this meant that some patients could not participate due to a lack of time available to stay at the clinic. Our analyses comparing the characteristics of patients consenting and not consenting suggested few important differences, except for men and those with arthritis who were more likely to participate.

In sensitivity analyses, blood pressure control was found not to be predictive of medication adherence. We defined BP control as +/− 140/90 mmHg, but for some individuals with co-morbid conditions such as diabetes, the definition of BP control may have been different [23].

### Comparison with existing literature

Adherence to antihypertensive medication has been widely studied, with estimates of non-adherence varying depending on the population studied and the methods used to measure medication adherence [24, 25]. Studies in different hypertensive populations using self-report questionnaires such as the Morisky medication adherence scale [12] have found rates of non-adherence of between 31.2% (hypertensive patients with co-morbidities), 45.2% (hypertensive patients), and 83.7% (hypertensive patients with uncontrolled blood pressure) [24]. Similarly, studies using objective biochemical measures (such as the LC-MS/MS method) have observed non-adherence rates of 25–42% [6, 17].

One previous study conducted in Irish primary care [21], approaching 453 patients with apparent treatment-resistant hypertension, found this method of determining adherence was feasible in 52% of patients approached. Of these, 24% were found to have partial non-adherence to antihypertensive medication and 2% were completely non-adherent. Another study in England including 228 diabetic patients attending primary care for an annual review, found non-adherence to antihypertensives was just 8%, with the highest of non-adherence rates (10%) seen for ACE inhibitors and angiotensin II receptor blockers [26].

To our knowledge, this is the first study conducted in primary care to assess medication adherence in the general hypertensive population. It reveals lower rates of non-adherence to antihypertensive medication (<5%) than previously reported [6, 17, 18, 21] and confirms previous observations about reduced adherence in patients taking multiple medications [5–7]. Differences in observed rates may be due differences in the populations studied. Participants in the present study generally had well-controlled blood pressure (mean 135/75 mm Hg; 62% controlled) and were not necessarily attending routine practice to address problems with their hypertension management. In contrast, previous studies have often enroled complex hypertensive patients from clinics where individuals have been referred due to suboptimal blood pressure control [6, 17, 18, 21]. We found non-adherence was most common for loop diuretic prescriptions, which may be related to the effect they can have on urinary urgency in older patients (i.e., they are less likely to be taken on days when an individual is expecting to be out of the house for a significant period of time). Loop diuretics may also be cleared from the kidneys quicker and non-adherence to these drugs may be linked to non-adherence associated with taking multiple medications [6].

### Implications for research and/or practice

The present findings highlight the discrepancy between patient self-reported adherence and actual medication-taking behaviour. However, given the low rates of non-adherence observed here, objective methods for measuring adherence (such as LC-MS/MS) may not be necessary for all patients in a primary care setting. These tests cost £40 per patient and are becoming increasingly popular in secondary care (currently 35 hypertension referral centres around the UK are using them). They are thought to be cost-effective as an intervention to improve medication adherence, particularly when targeted at groups most likely to be non-adherent [27]. The present study suggests that one such group to target may be those individuals prescribed multiple antihypertensive medications. Though not tested here, these tests might also be useful as part of the assessment of community patients with poor blood pressure control prior to intensifying therapy.

## Conclusions

Biochemical testing for antihypertensive medication adherence appears to be feasible in terms of hypertensive patient acceptance for those attending routine appointments in primary care. Non-adherence is generally low amongst such patients and so widespread testing is not recommended where resources are limited. In such situations, GPs may wish to focus testing on patients with poor control prescribed more antihypertensive medications who may be less likely to be adherent to therapy.

### Summary table

#### What is known on this topic

- New methods for identifying non-adherence exist using liquid chromatography-tandem mass spectrometry (LC-MS/MS) to detect a number of medications measurable in an individual’s urine.
- This approach has revealed high rates of previously unrecognised non-adherence to medication in hypertensive patients referred to specialist clinics in a secondary care setting.
- It is unclear whether the medication adherence rates seen in these complex patients are representative of those in the general population presenting in primary care.

#### What this study adds

- Biochemical testing for antihypertensive medication adherence is feasible in routine primary care, with two-thirds of patients able to provide a sample when approached opportunistically.
- Amongst those participants who were able to provide a urine sample, self-reported adherence to medication did not appear to be related to biochemically determined adherence.
- Overall, non-adherence to antihypertensive medication was very low (<5%), in contrast to previous studies using this approach, and therefore testing using LC-MS/MS is not recommended for widespread use in the community.

## Supplementary information

Supplementary appendix
