# Supplementary material for: Measuring adherence to antihypertensive medication using an objective test in older adults attending primary care: cross-sectional study
Source: J Hum Hypertens. 2021 Dec 7;36(12):1106–12. doi: 10.1038/s41371-021-00646-w (PMC7613908; doi:10.1038/s41371-021-00646-w)
Supplement: Supplementary file 1 — Supplementary appendix [file 41371_2021_646_MOESM1_ESM.docx]

**Measuring adherence to antihypertensive medication using an objective test in older adults attending primary care: cross-sectional study**

**Supplementary appendix**

James P Sheppard, *PhD*^a^ Ali Albasri, *DPhil* ^a^ Pankaj Gupta, *FRCP*^b,c^ Prashanth Patel, *FRCP*^b,c^ Kamlesh Khunti, *FMedSci*^b,c^ Una Martin, *FRCP*^d^ Richard J McManus, *FRCGP*^a^ FD Richard Hobbs, *FMedSci*^a^

^a^Nuffield Department of Primary Care Health Sciences, University of Oxford, Oxford, UK

^b^Department of Chemical Pathology and Metabolic Diseases, University Hospitals of Leicester NHS Trust

^c^Department of Cardiovascular Sciences, University of Leicester

^d^Birmingham Medical School, College of Medical and Dental Sciences, University of Birmingham

**Extended methods**

# *Study design*

This study used a prospective, observational cohort design, enrolling participants in primary care aged ≥65 years with hypertension and prescribed at least one blood pressure lowering medication. Patients attending the practice were asked to give informed consent and provide a urine sample for researchers to examine whether antihypertensive medications were present in their system. Anonymised data were collected for patients declining to participate in the study for comparison to those who did participate. Ethical approval for this study was given by South Central - Oxford A Research Ethics Committee (ref: 18/SC/0647). Detailed methods are given in the supplementary appendix.

##

## *Study Participants*

Consecutive patients aged ≥65 years with an electronic medical record coded diagnosis of hypertension and prescribed at least one blood pressure lowering medication were approached opportunistically. Participating general practices were located in the Thames Valley region of England. Antihypertensive medications were defined as any ACE inhibitor, angiotensin II receptor blocker, calcium channel blocker, thiazide and thiazide-like diuretic, loop diuretic, alpha-blocker, beta- blocker, aldosterone antagonist, vasodilator antihypertensive, centrally acting antihypertensive, direct renin inhibitor or adrenergic neurone blocking drug. Patients prescribed clonidine as monotherapy were excluded as this cannot be detected using the LC-MS/MS method.

Participating general practitioners (GPs) were asked to identify patients attending routine medication reviews or chronic disease management clinics meeting the eligibility criteria. Parallel clinics run by research nurses/facilitators were scheduled alongside these appointments, where interested patients could give informed consent and provide a urine sample. Consent and sample collection took place directly after each routine appointment to ensure the subsequent assessment of medication adherence was representative of an individual’s true drug taking behaviour. This approach was required to avoid participants changing their behaviour due to prior knowledge of participating in a research study.^1^

*Data collection*

For all patients approached to participate in the study, members of the care team extracted anonymised data from their electronic health record, detailing basic patient characteristics and medical history. These data were collected regardless of whether individuals went on to give consent for urine sample collection. Data included information relating to patient characteristics (age, sex, smoking status, height, weight), most recently recorded clinic blood pressure, medical history (including hypertension, cardiovascular disease, cerebrovascular disease, heart failure, diabetes, chronic kidney disease, atrial fibrillation, dementia, depression or arthritis), and all antihypertensive medications prescribed. Where data relating to blood pressure, height, weight or smoking status were missing in the electronic health record, these were taken by the research nurse at the study visit. . Prior to collecting the urine sample, all consenting participants were asked the question “*Have you taken all of your blood pressure pills today?*” and responses were categorised as *‘”all medications”, “some medications,”* or *“no medications.”*

*Urine sample collection and analysis*

Participants were informed from the outset that the study was investigating the feasibility of collecting urine samples and testing whether patients had taken all of their medications as prescribed. They were reassured that this information would remain entirely confidential and members of the care team would not be made aware of the results of the urine test. A 10 ml urine sample was collected in a plastic container immediately after informed consent had been obtained. Each sample was transferred from the clinic site to the laboratory at University Hospitals of Leicester at room temperature, via a post office next day delivery service.

All samples received by the laboratory were stored at -80°C and then batch analysed at the end of the study. Prior to LC-MS/MS analysis, samples were prepared by solvent extraction and a dilution technique using methods previously described.^2^ LC-MS/MS was performed to detect all antihypertensive drug classes, using an Agilent Technologies 1200 series High Pressure Liquid Chromatograph interfaced with an Agilent Technologies 6410 Triple Quad Mass Spectrometer fitted with a Jetstream electrospray (ESI) source using.^2^ This method screens for approximately 40 different antihypertensive drugs. Only members of the laboratory team had access to the study samples. All samples were destroyed at the end of the study, after the analysis had been completed.

##

## *Sample size calculation*

The study aimed to collect anonymised data from approximately 285 patients, gathering urine samples from at least 200 consenting participants. This assumed 70%^3^ of those approached would give informed consent for their samples to be collected and analysed, allowing a recruitment rate of 70% to be estimated with an accuracy of ±6% (95% confidence interval of 64% to 75%). Recruitment was permitted to continue until at least 200 participants had given informed consent. Based on previously observed rates of medication adherence using this method,^2^ recruitment of at least 200 participants was estimated to be sufficient to calculate a medication adherence of 75% to within ±7%.

*Statistical analysis*

The primary outcome of this study was to determine the proportion of patients attending a routine medication review or check-up in primary care (denominator population) who gave informed consent to provide a urine sample for analysis of medication adherence (numerator). We did not pre-specify feasibility criteria for this study, but instead use criteria from a previous study of urine analysis to measure medication adherence which defined the feasibility of this approach as ≥50% of patients agreeing to provide a sample and 95% of collected samples as being suitable for analysis.^4^ We examined participant characteristics predicting the likelihood of consent to provide a urine sample were explored using multivariable logistic regression.

Medication adherence was defined as a binary outcome; adherent patients were those in whom all prescribed antihypertensive medications were present their urine sample. Non-adherent patients were those in whom only some or none of their prescribed medications were detected in the urine sample. Adherence to medication was estimated using descriptive statistics, across the entire study population and sub-grouped by type of medication prescribed and whether blood pressure was controlled (±140/90 mm Hg).

Predictors of non-adherence to medication were explored with logistic regression, including age, sex, blood pressure, co-morbidities and number of antihypertensive medications as independent predictor variables. Sensitivity analyses were performed including blood pressure control (±140/90 mm Hg) in the model instead of continuous blood pressure variables. Analyses were undertaken for descriptive purposes only, so no attempt was made to reduce the model using selection methods. All analyses were undertaken using STATA version 16.0 (MP edition, StataCorp, College Station, Texas, USA) and data are presented as proportions with 95% confidence intervals (CI) or means ± standard deviation.

**References**

1. McCambridge J, Witton J, Elbourne DR. Systematic review of the Hawthorne effect: New concepts are needed to study research participation effects(). *Journal of Clinical Epidemiology.* 2014;67(3):267-277.

2. Tomaszewski M, White C, Patel P, et al. High rates of non-adherence to antihypertensive treatment revealed by high-performance liquid chromatography-tandem mass spectrometry (HP LC-MS/MS) urine analysis. *Heart (British Cardiac Society).* 2014;100(11):855-861.

3. Walters SJ, Bonacho dos Anjos Henriques-Cadby I, Bortolami O, et al. Recruitment and retention of participants in randomised controlled trials: a review of trials funded and published by the United Kingdom Health Technology Assessment Programme. *BMJ Open.* 2017;7(3).

4. Hayes P, Casey M, Glynn LG, et al. Measuring adherence to therapy in apparent treatment-resistant hypertension: a feasibility study in Irish primary care. *British Journal of General Practice.* 2019;69(686):e621-e628.
